# Performance of the Improved Priestley-Taylor Model for Simulating Evapotranspiration of Greenhouse Tomato at Different Growth Stages

**DOI:** 10.3390/plants11212956

**Published:** 2022-11-02

**Authors:** Xuewen Gong, Guokui Bo, Hao Liu, Jiankun Ge, Xiaoming Li, Shikai Gao

**Affiliations:** 1 Henan Key Laboratory of Crop Water Use, School of Water Conservancy, North China University of Water Resources and Electric Power, Zhengzhou 450045, China; 2 Key Laboratory of Crop Water Use and Regulation, Farmland Irrigation Research Institute, Chinese Academy of Agricultural Sciences, Ministry of Agriculture and Rural Affairs, Xinxiang 453002, China

**Keywords:** evapotranspiration, greenhouse tomato, improved Priestley-Taylor model, path analysis method

## Abstract

Mastering crop evapotranspiration (ET) and improving the accuracy of ET simulation is critical for optimizing the irrigation schedule and saving water resources, particularly for crops cultivated in a greenhouse. Taking greenhouse-grown tomato under drip irrigation as an example, two weighing lysimeters were used to monitor ET at two seasons (2019 and 2020), whilst meteorological factors inside the greenhouse were measured using an automatic weather station. Then the path analysis approach was employed to determine the main environmental control factors of ET. On this basis, an improved Priestley-Taylor (IPT) model was developed to simulate tomato ET at different growth stages by considering the influence of environmental changes on model parameters (e.g., leaf senescence coefficient, temperature constraint coefficient and soil evaporative water stress coefficient). Results showed that the average daily ET varied from 0.06 to 6.57 mm d^−1^, which were ~0.98, ~2.58, ~3.70 and ~3.32 mm/d at the initial, development, middle and late stages, respectively, with the total ET over the whole growth stage of ~333.0 mm. Net solar radiation (*R_n_*) and vapor pressure deficit (VPD) were the direct influencing factors of ET, whereas air temperature (*T_a_*) was the limiting factor and wind speed (*u*_2_) had a little influence on ET. The order of correlation coefficients between meteorological factors and ET at two seasons was *R_n_* > VPD > *T_a_* > *u*_2_. The IPT model can accurately simulate ET in hourly and daily scales. The root mean square error of hourly ET at four stages changed from 0.002 to 0.08 mm h^−1^ and daily ET varied from 0.54 to 0.57 mm d^−1^. The IPT coefficient was close to the recommended PT coefficient (1.26) when the average *T_a_* approaches 26 °C and LAI approaches 2.5 cm^2^ cm^−2^ in greenhouse conditions. Our results can provide a theoretical basis for further optimization of greenhouse crop irrigation schedules and improvement of water use efficiency.

## 1. Introduction

In recent years, the development rate of the greenhouse industry is very fast, leading to the global greenhouse area exceeding 4.05 billion ha [1]. In China, solar greenhouses have expanded 1.26 times from 2008 (0.25 million ha) to 2018 (0.59 million ha). Solar greenhouses provide suitable environmental conditions for crop growth, which is an important industry to ensure vegetable safety and promote local economic development. Crop evapotranspiration (ET) is the core of the soil–crop–atmosphere continuous circulation system, which not only determines water and heat transfer, but also affects energy storage and nutrient transport [2]. Therefore, mastering the crop ET and realizing accurate simulations are the key to optimize the irrigation schedule and realize water-saving, yield improvement, quality and efficiency [1,3].

In greenhouses, ET was affected by many environmental factors, e.g., solar radiation (*R_s_*), air temperature (*T_a_*), vapor pressure deficit (VPD) and wind speed (*u*) [2,4]. Generally, a higher *R_s_* will increase ET, and *R_s_* will affect the opening and closing state of stomata on leaves by influencing the changes of heat and humidity around the leaves. Studies have shown that *R_s_* was the main environmental factor affecting ET [2,4,5]. Comparatively, *T_a_* affects ET by increasing the kinetic energy of water molecules. The stomatal conductance of leaves increases with the increase of *T_a_*; thus, the transpiration becomes more intense. The increase of *T_a_* will also lead to the increase of VPD between the leaves and soil, thus enhancing evaporation and transpiration. Chen et al. (2013) [6] showed that higher *R_s_* and *T_a_* would often increase ET for greenhouse tomato, especially at the development stage. VPD reflects the difficulty of water in plants to enter the atmosphere, but due to the influence of external environmental conditions, the influence of VPD on ET for different crops will be different [7]. Due to the semi-enclosed structure of the solar greenhouse, the indoor *u* was usually lower and the effect of *u* on ET was generally negligible, but this phenomenon cannot be ignored under water stress conditions [4]. Previous studies have basically clarified the effects of environmental factors on ET; however, under high temperature and high humidity, it will seriously limit the water transfer process in crops, particularly inside the greenhouse. For example, a slight high temperature will lead to premature leaf failure of the tomato and transient water stress in the root zone of the crop, while extreme high temperatures would constrain the water uptake of the plant and limit its growth. However, how to consider the effects of these factors on ET and how to optimize the parameters when developing an ET model are still unknown.

As measuring ET is expensive and laborious, so ET is usually obtained by numerical simulation. The Penman–Monteith (PM) model, crop coefficient model and their modified forms are more frequently applied in greenhouses for simulating ET. For example, Yan et al. (2019) [8] improved the precision of the PM model for simulating cucumber transpiration by correcting canopy resistance; Morille et al. (2013) [9] simulated the transpiration of greenhouse balsamina by using the improved PM model, and indicated that the precision of the PM model could be better improved by modifying the canopy resistance according to the meteorological factors of different layer height positions; Qiu et al. (2013) [10] simulated the ET of greenhouse pepper for different planting densities by using a single crop coefficient model; Li et al. (2022) [11] indicated that the crop coefficient model was in agreement with the measured values; therefore, the crop coefficient model accurately estimated the daily ET of greenhouse-grown eggplant during each growth period. However, there are still some problems with the above models: (1) to improve the precision of the PM model, surface resistance parameters should be calculated accurately, but previous research on surface resistance only involve the crop canopy, whereas they rarely consider the soil surface, resulting in an overestimation of ET; (2) the dual crop coefficient method was often used to calculate daily ET, which not only requires one to accurately obtain crop coefficient at different stages, but also needs to accurately determine the reference ET in the greenhouse [12], resulting in many calculation processes that may increase the model error.

The Priestley-Taylor (PT) model, based on radiation theory, has a simple structure and high accuracy for simulating ET. The PT model has been applied in the field crops, such as maize [13], cotton [14] and wheat [15]. In the PT model, crop ET can be calculated carefully by multiplying the PT coefficient (*α*) and the equilibrium evaporation (ET_eq_), where ET_eq_ was determined by net radiation (*R_n_*), ground soil heat flux (*G*) and *T_a_*. Obtaining *α* accurately was the key to improving the precision of the PT model [13,15], as previous studies considered *α* as a constant (1.26) during the whole growth period [16,17]. However, some studies found that *α* was not only affected by the surrounding conditions, but also by other factors, such as plastic film coverage, canopy cover [18], soil available moisture [12] and leaf senescence [14]. Shao et al. (2022) [19] used the PT model to calculate the daily transpiration of tomatoes in a sunken heliostat in northern China, and Gong et al. (2021) [20] simulated the tomato ET in the solar greenhouse, indicating that establishing the *α* equation according to the environmental conditions was important. However, few studies have focused on this aspect in the greenhouse condition. Therefore, for the environmental characteristics of high temperature, high humidity and low wind speed in the greenhouse, we hypothesis that the *α* of tomatoes was affected by the combined influence of leaf senescence, temperature constraint and soil evaporative water stress, and this effect was different at the different crop growth stages. This paper carries out further in-depth research and innovation based on the paper from Gong et al. (2021) [21]. Therefore, whether the improved PT model can accurately simulate ET of tomatoes at different growth stages was the focus of this study.

## 2. Results

### 2.1. Meteorological Conditions

**Figure 1:**
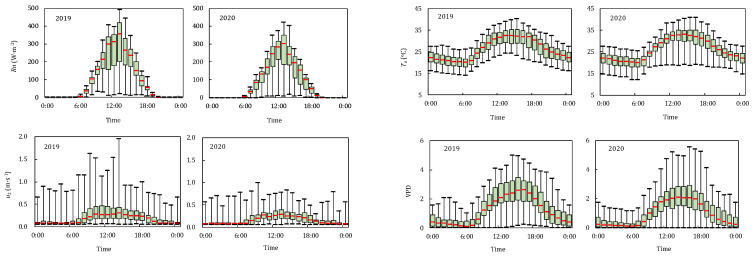
Daily variations of net radiation (*R_n_*), air temperature (*T_a_*), wind speed (*u*_2_) and vapor pressure deficit (VPD) in the greenhouse in 2019 and 2020. The upper and lower edges of each element in the box diagram are the maximum and minimum values, respectively. Box upper edge, lower edge and middle red line are the upper quartile, lower quartile and median of the data group, respectively.

Figure 1 shows the daily variation of meteorological factors, including net radiation (*R_n_*), air temperature (*T_a_*), wind speed (*u*_2_) and water vapor deficit (VPD). The data were the average values when the tomato entered the development stage in 2019 and 2020. Here, *R_n_* started from 6:00 then increased rapidly, the maximum value appeared between 13:00 and 14:00, and then gradually decreased. The difference of *R_n_* between the maximum and minimum was the biggest at 13:00 due to the influence of weather conditions. The maximum *R_n_* was 469.1 and 415.5 W m^−2^ in two seasons. *T_a_* and VPD were influenced by *R_n_*, but the maximum value lagged *R_n_* by 1 h. *T_a_* and VPD started to rise from 7:00 and gradually decreased after 16:00. The average *T_a_* and VPD varied from 19.7 to 25.7 °C and 0.2 to 1.2 kPa during evening time.

Due to the semi-enclosed characteristic of the greenhouse, the indoor *u*_2_ was lower, with the daily average *u*_2_ below 0.5 m s^−1^. However, the maximum and minimum values of *u*_2_ differ greatly, with the maximum differences of 1.9 and 0.8 m s^−1^ in 2019 and 2020, respectively, which was mainly influenced by the opening status of the vent and the outdoor wind speed.

### 2.2. Variations of Plant Height and LAI

**Figure 2:**
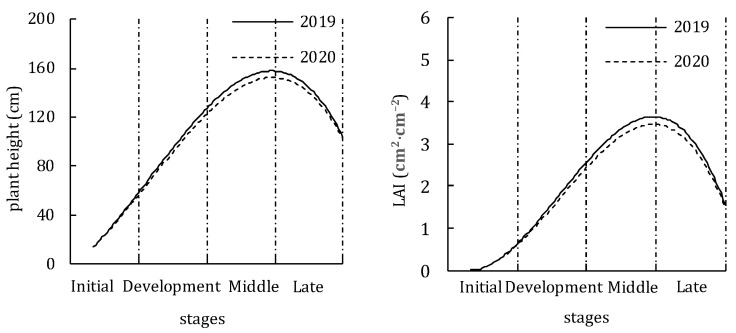
Variations of tomato plant height and LAI in 2019 and 2020.

Figure 2 shows the variations in plant height and LAI in two study years. The changes of plant height and LAI over the whole growth period showed a downward opening parabola. Plant height and LAI were small at the initial stage, lower than 50 cm and 0.8 cm^2^·cm^−2^. Entering the development stage, crop height and LAI increased rapidly, reached the maximum at the middle stage (~145 cm of crop height and 3.46 cm^2^·cm^−2^ of LAI) and showed a downward trend at the late stage due to leaf senescence. Furthermore, the curve of plant height and LAI was very close in two years, which may be related to the closer accumulated temperature inside the greenhouse at the two years.

### 2.3. Variations of ET

**Figure 3:**
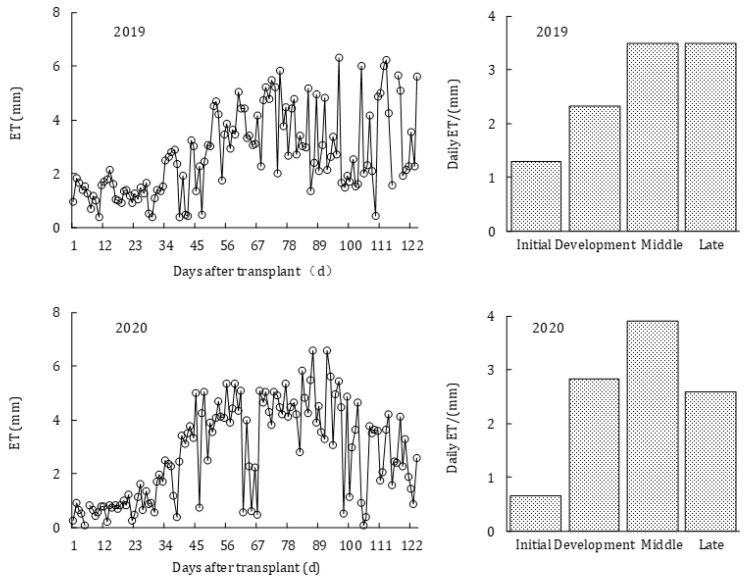
Variations of ET over the whole growth period and daily average ET at four growth stages in 2019 and 2020.

Figure 3 shows the variations of ET during the whole growth period and the daily average ET at four growth stages in 2019 and 2020. A tendency was shown for ET to increase first and then decrease, varying between 0.06 and 6.57 mm d^−1^ during the whole growth period. ET was lower at the initial stage, with the daily average values of 1.30 and 0.66 mm d^−1^ for two seasons. While ET gradually increased with the increase of *R_n_* and LAI, it reached the maximum value (3.49 and 3.90 mm d^−1^) at the middle stage. This was because the middle growth of tomato was just at the fruit setting period, where the demand for water was large. ET decreased less at the late stage because the tomato variety used in this study was the infinite growth type and *R_s_* was higher during this period. The average daily ET was not much different between two years, 2.73 and 2.79 mm d^−1^, respectively.

### 2.4. Main Meteorological Factors Affecting ET

The ET for greenhouse tomato was mainly affected by environmental conditions and the PT model was based on the theory of equilibrium evaporation, so the effect of environmental changes on model parameters should be given priority in the improvement. Here, the path analysis method was employed to express the relationship between hourly ET and meteorological factors (e.g., *R_n_*, *T_a_*, VPD and *u*_2_). The results are shown in Table 1, where ET had the highest correlation coefficients with *R_n_* (0.952 and 0.888) and the lowest with *u*_2_ (0.672 and 0.615). The magnitude of correlation coefficients between meteorological factors and ET in two seasons were ranked as *R_n_* > VPD > *T_a_* > *u*_2_ due to the direct and indirect effects of factors on ET. *R_n_* has the largest direct path coefficient on ET, followed by VPD and *u*_2_ was the smallest. The direct path coefficient of *T_a_* to ET was negative, indicating that *T_a_* was the limiting factor and mainly indirectly affects ET through VPD. The total indirect path coefficients of *T_a_*, VPD and *u*_2_ were all higher than the direct path coefficients, indicating that *R_n_* directly affected ET, while *T_a_*, VPD and *u*_2_ indirectly affect ET. In these meteorological factors, *R_n_* had the highest decision coefficient for ET in the two seasons, followed by VPD, indicating that *R_n_* and VPD were the main controlling environmental factors affecting ET.

### 2.5. Performance of the Improved PT Model

**Figure 4:**
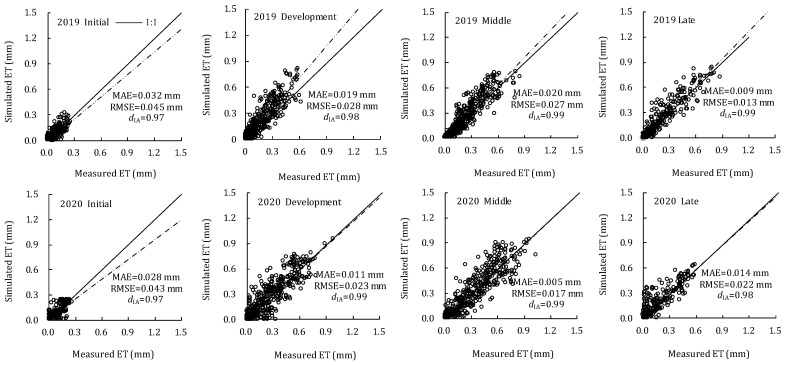
Comparison of measured and simulated hourly ET of greenhouse tomato at four growth stages in 2019 and 2020.

Figure 4 shows the comparison of measured and simulated hourly ET at four growth stages in 2019 and 2020. They show good agreement and were evenly distributed near the 1:1 line. The slope of the regression equation was close to 1.0, indicating that the simulated and measured ET were statistically similar and most of the variance can be explained by the IPT model. The IPT model can accurately simulate ET at hourly scales, with the mean absolute error (MAE) of ~0.03 mm at the initial stage, ~0.02 mm at the development stage and ~0.01 mm at the middle and late stages. The root mean square error (RMSE) fluctuates around 0.04, 0.03, 0.02 and 0.02 mm at the four growth stages, respectively, and the consistency index (*d*_IA_) varied between 0.98 and 0.99 during the whole growth stage.

**Figure 5:**
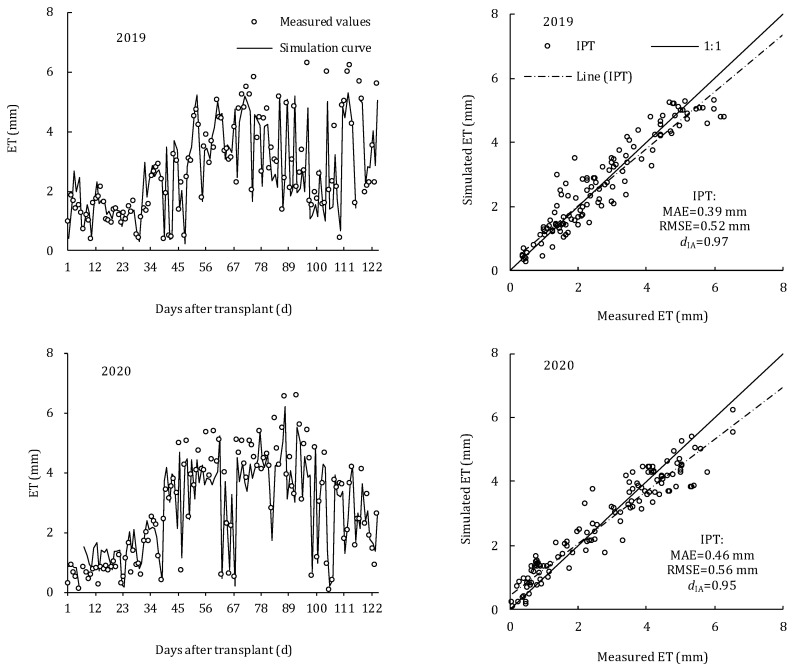
Comparison of measured and simulated daily ET of greenhouse tomato during the whole growth stage in 2019 and 2020.

The precision of the IPT model in simulating daily ET was also verified (Figure 5). The measured ET values in two seasons were also distributed around the simulated curve, and the regression equation between the measured and simulated ET could be fitted as y = 0.94x. Only 2.38% and 2.68% of ET were underestimated in 2019 and 2020, respectively. The IPT model had lower RMSE and higher *d*_IA_ in simulating daily-scale ET, with RMSE of 0.52 and 0.56 mm d^−1^, and *d*_IA_ of 0.97 and 0.95, in 2019 and 2020, respectively. Therefore, the IPT model can well simulate the daily and hourly ET of tomato under drip irrigation in greenhouses.

### 2.6. Variation of Improved PT Coefficient and Its Influencing Factors

**Figure 6:**
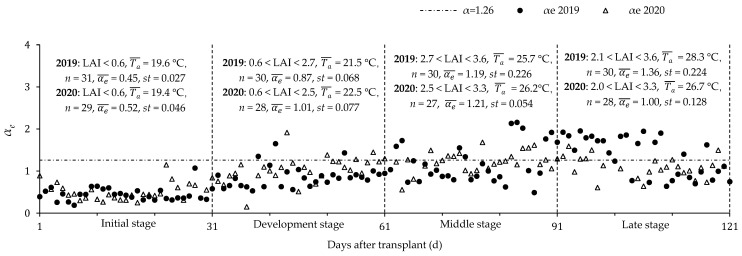
Variations of improved Priestley-Taylor coefficient (*α_e_*) during the whole growth stage in 2019 and 2020. LAI is the leaf area index, $\bar{T_{a}}$ is the mean air temperature, $\bar{\alpha_{e}}$ is the mean of *α_e_*, *st* is the variance of *α_e_*.

The main factors that affect the improved PT coefficient (*α_e_*) in the greenhouse include *T_a_*, LAI and soil water stress. Due to the needs of experimental management, greenhouse tomatoes would experience a water deficit at the initial stage, so the soil evaporative water stress coefficient (*f_sw_*) was equal to *S_e_* (*S_e_* can be calculated by Equation (11)). After entering the development stage, greenhouse tomatoes were not affected by soil water stress due to the increase in irrigation frequency, so *f_sw_* was taken as 1. Figure 6 shows the variations of the *α_e_* during the whole growth period, and the average *T_a_* ($\bar{T_{a}}$) and LAI at the four growth stages. At the initial stage (LAI < 0.6; $\bar{T_{a}}$ < 20 °C), the *T_a_* was lower than the optimal growth temperature of tomatoes (26 °C) and the *α_e_* varied between 0.45 and 0.52, that was 58.7% to 64.3% lower than the recommended PT coefficient (1.26). The *α_e_* gradually increased with the growth stage and reached the maximum value at the middle and late stages (LAI > 1.0; $\bar{T_{a}}$ > 25 °C), where the maximum value of *α_e_* exceeded 2.0 and the standard deviation varied between 0.054 and 0.226. The *α_e_* fluctuated around the distribution 1.26 when $\bar{T_{a}}$ was close to 26 °C during the middle and late stages, indicating that *α_e_* can be taken as 1.26 when the air temperature inside the greenhouse fluctuated around the optimal growth air temperature.

## 3. Materials and Methods

### 3.1. Experimental Site

The experiment was carried out from March to July in 2019 and 2020 at the Agro-ecological Experimental Station, which was located in Xinxiang city, Henan province of China (35.14 N, 113.78 E, altitude 78 m). The site was in the temperate continental monsoon climate zone, with the average air temperature of 23.8 °C during the experiment. The coldest month was in January with a mean value of 0.7 °C, while the hottest month was in July with an average value of 27.1 °C. The average annual precipitation was ~573 mm, which mainly concentrated in July to September.

The solar greenhouse used in the experiment was 60 m long, 8.5 m wide and 3.9 m high of the ridge. A brick structure with inlaid insulation was used on the north and hill walls, whilst the steel frame structure was covered with polyethylene film on the top. To improve the thermal insulation of greenhouse, 0.025 m thick thermal insulation cotton quilt was laid on the polyethylene film to ensure the temperature required for the growth of tomato seedlings at night. The temperature, humidity and wind speed inside the greenhouse were controlled by natural vents on the north wall, roof and south wall. Soil texture at depth of 0–1.0 m was a silt loam (16.9% clay, 76.7% silt and 6.4% sand) with bulk density of 1.49 g cm^−3^. The field capacity was 0.32 cm^3^ cm^−3^ and wilting point (*θ_w_*) was 0.09 cm^3^ cm^−3^.

### 3.2. Experimental Design

A local leading variety of tomato (*Solanum lycopersicum*, c.v. Jinding) was used and the seedlings were transplanted on the 11 March 2019 and 4 March 2020. The plots were 8.0 m long and 2.2 m wide, with 20 groups. The plot was planted in wide (0.65 m) and narrow (0.45 m) rows in a north–south direction with the planting density of 5.7 plants m^−2^. A drip irrigation system was used with one drip line per crop row and the spacing of emitter was the same as plant spacing. Pressure-compensated emitters with a discharge rate of 1.1 L h^−1^ were used. To ensure the survival of seedlings, 20 mm of water was irrigated by a drip irrigation system after transplanting. Entering the development stage, the irrigation frequency was determined based on the measurement of an evaporation pan (20 cm in diameter and 11 cm in depth), which was located at a height of 20 cm above the canopy. When cumulative evaporation (*E_pan_*) reached 20 ± 2 mm, the irrigation water amount of 0.9 *E_pan_* was irrigated [4,22]. The trial base fertilizer was 112 kg hm^−2^ urea (containing N 46%), 150 kg·hm^−2^ potassium sulfate (containing K_2_O 50%) and 120 kg hm^−2^ calcium superphosphate (containing P_2_O_5_ 14%). Thereafter, when the fruit of the first ear grew to the size of a walnut, fertilizer was applied with irrigation water. Each fertilization consisted of 18.8 kg hm^−2^ urea and 25 kg hm^−2^ potassium sulfate for six times. Four growth stages were divided, including initial stage (from transplanting to reaching approximately 10% ground cover, 12 March–2 April 2019 and 4–25 March 2020), development stage (from approximately 10% ground cover to the ground being effectively fully covered, 3 April–11 May 2019 and 26 March–5 May 2020), middle stage (from when the ground was effectively fully covered to when the fruit begins to ripen, 12 May–20 June 2019 and 6 May–16 June 2020) and late stage (from the start of fruit ripening to the end of the trial, 21 June–13 July 2019 and 17 June–7 July 2020). Daily management such as pollination, pest and disease control and topping and other agronomic measures were the same.

### 3.3. Measurements

The meteorological factors inside the greenhouse were monitored using an automatic weather station located in the middle greenhouse, which included a net radiation sensor (NR LITE2, Kipp & Zonen, Delft, The Netherlands), a temperature and humidity sensor (CS215, Campbell Scientific, Inc., Logan, UT, USA) and wind speed sensor (Wind Sonic, Gill, UK) at a height of 2.0 m above the ground surface. Two heat flux plates (HFP01, Hukseflux, The Netherlands) were used to measure soil heat flux (*G*) 5 cm below the ground surface between two rows. All data were collected at 5 s intervals and averages were calculated at 30 min and stored in a data collector (Campbell Scientific Inc., Logan, UT, USA). The mean net radiation, air temperature, wind speed and water vapor deficit in the greenhouse at four growth stages in 2019 and 2020 are listed in Table 2.

In this study, the hourly ET was measured by two weighing lysimeters, which were made of steel with a rectangular shape (length × width × depth = 1.0 m × 1.0 m × 1.2 m). Lever principle with high precision electronic scales were equipped in the weighing lysimeters. The soil texture inner weighing lysimeter was the same as the field, and the bottom was filled with a 10 cm layer of coarse gravel to facilitate drainage. Six tomato plants were transplanted in the same way as the field. ET data were measured every 5 min and calculated every 1 h, then the average values were stored in the mini computer.

Volumetric water content at 5 and 10 cm soil layer were measured continuously using three ECH_2_O soil moisture sensors (5TE, Decagon Devices, Inc., Pullman, WA, USA); these data were recorded every 30 min and stored in the EM50 collector (Decagon Devices, Inc., Pullman, WA, USA). The saturated water content from 0 to 10 cm was 0.32 cm^3^ cm^−3^, which was measured by the cutting ring method. The wilting point from 0 to 10 cm was 0.09 cm^3^ cm^−3^, which was measured by the sealed cultivation method.

The extinction coefficient (*k*) was measured by using two photosynthetic active radiation (PAR) sensors (LI-COR, Lincoln, NE, USA), which were mounted at 0.2 m above the tomato canopy and ground surface, respectively. The measurement was monitored from 6:00 to 19:00 on sunny days every week. A LI-1400 data logger was used to collect PAR data at a 15 min interval. The *k* can be calculated as follows:

(1) $$k=-\frac{\ln\left({\mathrm{PAR}}_{s}/{\mathrm{PAR}}_{o}\right)}{\mathrm{LAI}}$$

where PAR_s_ and PAR_o_ were the photosynthetic active radiation reaching the ground and canopy surface, respectively (umol (m^2^·s)^−1^).

Plant height was measured with a straightedge whilst leaf area was determined by summing the rectangular area (length × maximum width) of each leaf multiplied by a reduction factor of 0.64 [7]. Nine replications were manually measured every week. Leaf area index (LAI) was defined as the ratio of plant leaf area to unit soil surface area. The piecewise cubic Hermite interpolating polynomial of MATLAB software was adopted to calculate the daily LAI (MathWorks Inc., Natick, MA, USA). Canopy coverage was determined by vertical photographing from top to bottom, which was processed by CAN-EYE software (https://www6.paca.inrae.fr/can-eye/, accessed on 1 October 2022).

### 3.4. Improved Priestley-Taylor Model

The PT model was the simple form of the Penman–Monteith equation, which is based on the equilibrium evaporation [23]:

(2) $$\lambda \mathrm{ET}=\alpha \;\cdot {\;\lambda \mathrm{ET}}_{\mathrm{eq}}=\alpha \frac{\Delta }{\Delta +\gamma }\left(R_{n}\;-\;G\right)$$

where λET is the latent heat flux (W m^−2^); λET_eq_ is the equilibrium evaporation (W m^−2^); *α* is the bulk PT coefficient; *Δ* is the slope of field capacity water vapor pressure versus *T_a_* curve (kPa K^−1^); *γ* is the psychometric constant (kPa K^−1^); *G* is the ground soil heat flux (W m^−2^).

Generally, λET was the sum of soil evaporation partial energy (λE) and crop transpiration partial energy (λT), and λE and λT can be calculated as follows:

(3) $$\{\begin{array}{l} {\lambda E=\alpha }_{s}\frac{\Delta }{\Delta +\gamma }(R_{\mathrm{ns}}\;-\;G)\\ {\lambda T=\alpha }_{c}\frac{\Delta }{\Delta +\gamma }R_{\mathrm{nc}}\;\;\;\;\;\;\;\;\;\;\; \end{array}$$

where *R_ns_* and *R_nc_* are the received energy by soil and canopy surface (W m^−2^), respectively; *α_s_* and *α_c_* are the coefficients for λE and λT, respectively. *R_ns_*, *R_nc_*, *α_s_* and *α_c_* are determined as follows [23]:

(4) $$\{\begin{array}{l} R_{\mathrm{ns}}=\tau \cdot R_{n}\\ R_{\mathrm{nc}}=(1\;-\;\tau )\cdot R_{n}\\ \tau =R\cdot \exp(-\mathrm{kLAI}) \end{array}$$

(5) $$\{\begin{array}{l} \alpha_{s}{=f}_{\mathrm{sw}}\cdot \alpha_{s0}\\ \alpha_{c}{=(1\;-\;f}_{s})\cdot f_{t}\cdot \alpha_{c0} \end{array}$$

where *τ* is the fraction of radiation transmission reaching the soil surface, and varied with LAI; *f_sw_* is the water stress coefficient of soil evaporation, given as Equation (6) [24]; *α_s_*_0_ and *α_c_*_0_ are the IPT coefficients for soil and canopy, respectively, under energy-limited conditions, given as Equations (7) and (8) [25]; *f_s_* and *f_t_* are the leaf senescence index and plant growth limiting factor, respectively, given as Equations (9) and (10) [26,27].

(6) $$f_{\mathrm{sw}}=\{\begin{array}{ll} 1.0 & S_{e}\geq 0.75\\ S_{e} & S_{e}<0.75 \end{array}$$

(7) $$\alpha_{s0}=\{\begin{array}{c} 1.0\;\;\;\;\;\;\;\;\;\;\;\;\;\;\;\;\tau \;\leq {\;\tau }_{c}\\ \alpha_{0\;}-\frac{{(\alpha }_{0}\;-\;1)(1\;-\tau )}{{1\;-\;\tau }_{c}}{\tau \;>\;\tau }_{c} \end{array}$$

(8) $$\alpha_{c0\;}=\frac{\alpha_{0\;}{-\;\alpha }_{s0}\cdot \tau }{1\;-\;\tau }$$

(9) $$f_{s}=0.05\exp(\frac{\mathrm{CDC}}{0.98}t\;-\;1)$$

(10) $$f_{t}=\exp\left[-{(T_{a}{/T}_{\mathrm{opt}}\;-\;1)}^{2}\right]$$

where *α*_0_ is the recommended PT coefficient (1.26) [23]; *τ_c_* is a critical value of *τ* when canopy coverage reaches the maximum, 0.55 in our study [28]; CDC is the canopy decline coefficients, 0.08 for tomato grown in a greenhouse [29]; *t* is the data when the canopy starts to be senescent (d); *T_opt_* is the optimum temperature for tomatoes (°C), 26 °C in our study [29]; *S_e_* is the saturation of effective soil moisture in the top 0.1 m soil layer, can be determined as:

(11) $$S_{e}=(\theta \;-\;\theta_{w})/(\theta_{s}\;-\;\theta_{w})\;$$

where *θ*, *θ_s_* and *θ_w_* are the measured, saturated and wilting water content in the top 0.1 m of soil depth (cm^3^ cm^−3^), respectively.

To derive an analytical expression of the improved PT coefficient (*α_e_*), *G* should be described as a function of *R_ns_*, given as:

(12) $$G=f_{G}\;\cdot \;R_{\mathrm{ns}}$$

where *f_G_* is the fraction of *G* to *R_ns_*, 0.45 in our study, which calculated based on the measured *G* and calculated *R_ns_*. The expression of *α_e_* can be obtained by combining Equations (2)–(12):

(13) $$\alpha_{e}=\frac{f_{\mathrm{sw}}\cdot \alpha_{s0}{(1\;-\;f}_{G})\cdot (-k\cdot {\mathrm{LAI})+(1\;-\;f}_{s})\cdot f_{t}\cdot \alpha_{c0}\cdot [1\;-\;\exp\left(-k\cdot \mathrm{LAI}\right)]}{1\;-\;\exp(-k\cdot \mathrm{LAI})\cdot f_{G}}$$

### 3.5. Model Evaluation

The performance of the IPT model for estimating ET were evaluated by three commonly used statistical indicators. These indicators were root mean square error (RMSE), mean absolute error (MAE) and index of agreement (*d*_IA_), defined as follows:

(14) $$\mathrm{RMSE}={\left[\frac{\sum_{i=1}^{n}{\left(O_{i}\;-\;P_{i}\right)}^{2}}{n}\right]}^{0.5}$$

(15) $$\mathrm{MAE}=\frac{1}{n}\sum_{i=1}^{n}|O_{i}\;-\;\bar{P}|$$

(16) $$d_{\mathrm{IA}}=1.0\;-\;\frac{\overset{n}{\underset{i=1}{\sum }}{\left(O_{i}\;-\;P_{i}\right)}^{2}}{\overset{n}{\underset{i=1}{\sum }}{\left(|P_{i}\;-\;\bar{O}|+|O_{i}\;-\;\bar{O}|\right)}^{2}}$$

where *O_i_* and *P_i_* are the measured and calculated values, $\bar{P}$ and $\bar{O}$ are the average values, respectively; *n* is the number of measurement values. The accuracy of the model was highest when RMSE and MAE were close to 0 and *d*_IA_ was close to 1.0.

## 4. Discussion

### 4.1. Variation of ET and Its Influencing Factors

Water was the main component for all physiological activities and the synthesis of dry matter in crops. The water demand of crops varies at different growth stages, and more that 90% of water was used for plant transpiration. The ET of greenhouse tomatoes under drip irrigation was small at the initial stage and then gradually increased with the increase of *R_n_* and LAI at the development stage. Entering the middle stage, the fruit expands gradually, which was a critical period for water demand, resulting in the increase of ET. The LAI decreased slightly at the late stage due to leaf senescence and ET decreased slightly at this time, but ET was still at a high level. The average daily ET varied from 0.06 to 6.57 mm d^−1^ during the whole growth period. There was no significant difference in ET between two seasons, with the values of 2.73 and 2.79 mm d^−1^, respectively, which was similar to the results of Liu et al. (2013) [22] and Ge et al. (2021) [30], but lower than the field ET with border irrigation [7]. This may be caused by the influence of greenhouse conditions, e.g., soil moisture, high humidity, wind speed and solar radiation.

Crop ET in greenhouses was influenced by many meteorological factors, such as *R_n_*, *T_a_*, VPD and *u*_2_, particularly for *R_n_* and VPD. *R_n_* was the main control factor affecting the ET, which can not only influence stomatal opening, but also determine the distributions of *T_a_* and RH surrounding the plant leaf. In addition, *R_n_* can change the leaf surface temperature to increase VPD and promote plant transpiration, which was similar to the results of Qiu et al. (2019) [15] on greenhouse pepper and Demrati et al. (2007) [31] on greenhouse banana. VPD was another important factor affecting ET and reflects the difficulty of the water entering the atmosphere. The direct path coefficients for ET were 0.35 and 0.29 in 2019 and 2020, respectively, indicating that the ET of greenhouse tomato was significantly affected by VPD. However, Granier et al. (1996) [32] suggested that VPD was the main environmental factor affecting plant transpiration in tropical rainforest areas, rather than *R_n_*, which was possibly due to the high humidity in this site. *T_a_* affects ET by increasing the kinetic energy of water molecules. The higher *T_a_* is, the greater stomatal conductance of leaves is, leading to the enhancement of transpiration. The increase of *T_a_* will also lead to the increase of VPD differences between inside and outside the leaves and the soil, thus enhancing evaporation and transpiration. In our study, a negative direct flux coefficient for *T_a_* on ET was conducted, indicating that *T_a_* was a limiting factor for ET, which may be caused by the frequent high *T_a_* during the noon hours in the greenhouse. Hayat et al. (2021) [33] suggested that the effect of *T_a_* on transpiration was stronger than VPD for Salix psammophila in a semiarid area of northwest China, which may be related to possible physiological changes in plants in response to high temperature stress. The effect of *u*_2_ on ET was small but not negligible. Air flow can take away excess water vapor from the air in a greenhouse, which increases the water vapor difference between inside and outside the tomato leaves and facilitates transpiration in tomatoes. The studies of Gong et al. (2022) [34] indicated that appropriate ventilation and water management strategies are beneficial to boost crop yield and quality in greenhouses, and an appropriate ventilation rate (0.8–1.0 m s^−1^) can improve leaf area index, stomatal conductance, transpiration rate and photosynthetic rate, hence improving yield.

### 4.2. Evaluation of Improved Priestley-Taylor Model

The precision of the PT model can be improved by modifying the Priestley-Taylor coefficient, which was comprehensively affected by meteorological factors (*T_a_*, humidity and *u*_2_), crop morphology (LAI and canopy coverage) and soil factors (soil moisture and plastic film coverage) [16]. In our study, the PT model was improved by introducing several key parameters affecting ET, for instance, leaf senescence coefficient and temperature constraint coefficient used to restrict the plant transpiration, and evaporation of soil water stress coefficient used to restrict the soil evaporation. The IPT model had better performance in simulating ET at different growth stages, with a lower root mean square error (RMSE) and higher consistency index (*d*_IA_) at hourly and daily scales, indicating that the IPT model had a higher simulation accuracy at different scales. In addition, the accuracy at the development, middle and late stages was better than the initial stage, which may be due to the fact that the water consumption of tomatoes under drip irrigation in greenhouses was mainly caused by soil evaporation at the initial stage, while the PT model had a low simulation accuracy on soil evaporation. As oil evaporation was influenced by surface soil moisture, it was difficult for the PT model, an energy-driven radiation-based model, to accurately simulate soil evaporation. In addition, there were two other reasons for simulation errors: (1) the model ignored the correlation between crop physiological characteristics and canopy (or LAI) and surface soil moisture; (2) the uneven soil surface moisture under drip irrigation will lead to the calculation deviation of soil evaporative water stress coefficient [35].

At present, there were few studies on the PT coefficient and its improved form in greenhouses. Shao et al. (2022) [19] used the PT model to calculate the daily transpiration of tomatoes in a sunken heliostat in northern China and pointed out that the PT model had large errors when plants were subjected to high temperatures and water stress. Gong et al. (2021) [20] also believed that the PT coefficient should be modified in combination with environmental changes due to the semi-closed characteristic of a greenhouse, whilst the ET of tomatoes in greenhouses was seriously underestimated by using the unimproved PT model. In this study, the greenhouse tomato ET was divided into four stages of research, and *α* was modified by considering the effects of leaf senescence, air temperature and soil water stress. We found that the modified PT coefficient in our study was dynamic during the whole growth period. However, Valdes-Gomez et al. (2009) [36] suggested that the PT coefficient taken at a value of 1.12 could simulate ET of tomato at the middle and late stages in the greenhouse. This difference may be due to differences in local climatic conditions. Studies on the PT model and its modifications were first conducted in the field condition, for example, Qiu et al. (2019) [15] improved the PT model by introducing the leaf senescence coefficient and soil stress index, which improved the simulation accuracy of ET in rice–wheat rotations. Ding et al. (2013) [13] concluded that the correction of PT coefficients by LAI, soil moisture, plastic film coverage and leaf senescence coefficient could improve the accuracy of ET in the hourly and daily scale of mulched drip irrigation maize. These studies focused on the effects of leaf senescence coefficient and soil stress index on ET in the improvement of the PT model, which was different from our study. We believed that the temperature constraint coefficient in greenhouse conditions was the key to affect the accuracy of the PT model. Although the modified PT model can better simulate ET in greenhouse tomato, the simulated ET still has some deviation from the measured ET, which may be caused by the edge effect of the lysimeter itself and the limitation of the horizontal diffusive movement of soil water [37].

## 5. Conclusions

Considering that the Priestley-Taylor model is the simple form of the Penman–Monteith model based on the equilibrium evaporation, we analyzed the variation pattern of ET and conducted a path analysis on the main control factors of ET in greenhouse tomato. We found that the direct path coefficient of solar radiation to ET was the highest, which was ~0.760, indicating that the improvement of the PT model was feasible. Since tomato cultivation will experience soil water deficit at the initial stage, high temperature stress at the mid-late stage and leaf senescence at the late stage, thus the leaf senescence coefficient, temperature constraint coefficient and soil evaporative water stress coefficient were mainly introduced in the improved PT model. The performance of the improved PT model was excellent and was able to accurately simulate the ET of greenhouse tomato at different growth stages, both in the daily and hourly scales, particularly at the middle stage with the highest simulation accuracy. The dynamic changes of the modified PT coefficient (*α_e_*) during the whole growth period were also analyzed and we found that the difference between *α_e_* and the recommended value (1.26) was large at the initial stage, and gradually decreased after entering the development stage. When the average air temperature approached ~26 °C and LAI approached 2.5 cm^2^ cm^−2^, it could be considered that *α_e_* = 1.26. The results of our study can provide a reference for the selection of precise irrigation indexes.

## Figures and Tables

**Table 1:** Path analysis results of ET, net radiation *R_n_*, air temperature *T_a_*, vapor pressure deficit VPD and 2 m high wind speed *u*_2_ of greenhouse tomato under drip irrigation.

Year	Factors	Correlation Coefficient	Direct Path Coefficient	Indirect Path Coefficient					Decision Coefficient
				Total Indirect Path Coefficient	*R_n_*	*T_a_*	VPD	*u*_2_	
2019	*R_n_*	0.952	0.744	0.208		−0.082	0.257	0.033	0.863
	*T_a_*	0.811	−0.108	0.919	0.564		0.320	0.035	−0.187
	VPD	0.833	0.353	0.481	0.541	−0.098		0.038	0.463
	*u*_2_	0.672	0.056	0.416	0.445	−0.067	0.038		0.072
2020	*R_n_*	0.888	0.775	0.256		−0.020	0.218	0.058	0.776
	*T_a_*	0.731	−0.026	0.933	0.605		0.265	0.064	−0.039
	VPD	0.751	0.291	0.630	0.581	−0.024		0.073	0.352
	*u*_2_	0.615	0.112	0.461	0.403	−0.015	0.073		0.125

Note: All meteorological factors are hourly data during the ventilation period (9:00–16:00).

**Table 2:** Average of net radiation (*R_n_*, W m^−2^), air temperature (*T_a_*, °C), wind speed (*u*_2_, m·s^−1^) and water vapor deficit (VPD, kPa) at four growth stages in 2019 and 2020.

Year	Growth Stages	*R_n_*	*T_a_*	VPD	*u*_2_
2019	Initial	114.81	19.19	1.031	0.118
	Development	125.65	21.26	0.716	0.120
	Middle	149.92	26.39	1.419	0.102
	Late	148.44	28.23	1.296	0.089
	Whole growth stage	135.80	23.92	1.106	0.101
2020	Initial	103.32	18.88	0.791	0.114
	Development	131.53	20.82	0.700	0.112
	Middle	142.53	25.48	1.172	0.145
	Late	154.09	27.03	0.710	0.138
	Whole growth stage	138.11	23.07	0.877	0.128

Note: *R_n_* is the average data of 6:00–18:00, other meteorological factors are the average values in the whole day.

## Data Availability

Not applicable.
